# Aging, frailty, and their effects on motor performance: evidence from kinematic analysis

**DOI:** 10.1007/s10072-025-08092-z

**Published:** 2025-03-18

**Authors:** Martina De Riggi, Giulia Paparella, Antonio Cannavacciuolo, Martina Salzillo, Filippo Nuti, Ludovica Quarta, Daniele Birreci, Davide Costa, Luca Angelini, Marco Canevelli, Matteo Bologna

**Affiliations:** 1 Department of Human Neurosciences, Sapienza University of Rome, Rome, Italy; 2 IRCCS Neuromed, Pozzilli (IS), Italy; 3 Department of Clinical Internal, Anesthesiologic and Cardiovascular Sciences, Sapienza University of Rome, Rome, Italy

**Keywords:** Aging, Frailty, Motor control, Kinematic, Finger-tapping

## Abstract

**Introduction:**

Aging is commonly linked to motor impairment. However, the relationship between motor performance across age groups and frailty remains unexplored through objective analysis.

**Objective:**

To kinematically evaluate motor performance in older and younger adults and investigate its potential connection to frailty.

**Methods:**

We included 65 healthy subjects (40 females, age range 46–83 years). We used kinematic techniques to study finger-tapping and measure several movement parameters, i.e., number of movements, movement rhythm, amplitude and velocity, as well as progressive reduction in amplitude and velocity during movement repetition (sequence effect). The frailty status was evaluated using a 40-item Frailty Index (FI). We also evaluated cognitive functions, using the Mini Mental State Examination (MMSE) and the Frontal Assessment Battery (FAB). We tested possible relationships between clinical and kinematic data using Spearman’s correlation.

**Results:**

A key finding was a significant difference in movement velocity between younger and older adults, with the latter exhibiting lower values (*P* = 0.014). Accordingly, age significantly correlated with movement velocity (*ρ* = -0.335, *P* = 0.037). Among older adults, movement velocity was also found to correlate with frailty (*ρr* = -0.297, *P* = 0.033), thus indicating that greater frailty is associated with more impaired motor performance.

**Conclusions:**

The relationship between the age-related slowed movement execution and frailty suggests that motor performance may serve as a sensitive indicator of physical vulnerability in aging populations.

**Supplementary Information:**

The online version contains supplementary material available at 10.1007/s10072-025-08092-z.

## Introduction

Population aging, driven by longer life expectancy and declining birth rates, is becoming an increasingly important issue worldwide. Italy, one of the world's oldest countries, has 23% of its population over 65 and 7.5% over 80 [1]. Aging is characterized by physiological brain changes such as atrophy, small vessel disease, and protein aggregation [2]. At the cellular level, mitochondrial dysfunction, oxidative stress, and neurotransmitter dysregulation (e.g., GABA, acetylcholine, dopamine) alter brain activity, leading to excitatory imbalances and reduced synaptic plasticity [2].

From a clinical standpoint, aging is often characterized by motor impairments [3–6] and cognitive decline, particularly affecting language, visuospatial, and executive functions [7]. Motor deficits in older adults include slowed movements, impaired coordination, and balance issues. A commonly used assessment of fine motor abilities involves repetitive finger-tapping, which relies on the proper functioning of the corticospinal tract, basal ganglia, cerebellar motor circuits, and proprioceptive systems [8–10]. Various studies have shown a decrease in the maximum finger-tapping repetition rate among older adults, highlighting the concept of age-related motor slowing [5, 6, 11–13]. These findings suggest that older individuals take more time to plan and initiate movements and exhibit less fluid, more segmented motion patterns compared to younger adults. However, the objective quantification of motor impairments in older adults using kinematic methods remains largely underexplored. Addressing this issue is important for distinguishing between age-related motor changes and conditions like Parkinson's disease (PD) or other neurodegenerative disorders, which pose significant diagnostic challenges [11].

Frailty, a condition of increased vulnerability to adverse health outcomes, is closely linked to functional decline in older adults. Clinically, frailty is a medical syndrome characterized by the reduction of the individual’s homeostatic reserves that leads to an increased vulnerability to stressors and to a higher risk for adverse outcomes [14–16]. In recent years, research has explored the impact of frailty on various pathological conditions affecting the central nervous system, including PD, Alzheimer’s Disease (AD), cognitive impairment, and psychiatric disorders [17–19]. Motor impairment, especially in the form of reduced physical performance and motor vigor, is another one of the earliest and most recognizable phenotypic manifestations of frailty [20]. Frailty further exacerbates motor impairment by diminishing the body’s ability to adapt to physical stressors, such as illness or injury, creating a vicious cycle where motor decline worsens frailty, and frailty in turn amplifies motor dysfunction. However, the specific impact of frailty on motor performance in otherwise healthy older individuals remains understudied.

In the present study, we aim to investigate the relationship between aging, frailty, and motor alterations in healthy subjects [8, 21, 22]. To this aim, we tested repetitive finger-tapping, a widely employed and sensitive clinical assessment tool [8, 12, 23]. Using kinematic techniques, we objectively measured various movement parameters, including number of movements, movement rhythm, velocity and amplitude, progressive reduction of amplitude and velocity during movement repetition, i.e. the sequence effect [9, 11, 23–26]. We then analyzed potential correlations between demographic and clinical data and kinematic findings. The findings from this study may provide valuable insights into the broader implications of aging on motor function.

## Methods

### Participants

The study was conducted at the Department of Human Neurosciences, Sapienza, University of Rome. We consecutively enrolled 65 subjects aged > 18 (40 females, mean age ± 1 standard deviation—SD: 65.40 ± 8.80 years). None of the participants had a history of neurological or psychiatric disorders, nor were they taking any medications affecting the nervous system activity. All of them were right-handed, as evaluated by the Handedness Questionnaire [27]. We collected a detailed medical history and performed a physical and neurological examination. We assessed the participants’ global cognitive performance by the means of the Mini Mental State Examination (MMSE) [28], and the Frontal Assessment Battery (FAB) [29]. Raw scores were adjusted for demographic variables (i.e., age and education). All subjects gave their written informed consent to the study. The experimental procedures, which adhered to the Declaration of Helsinki regulations and to international safety guidelines, were approved by the local institutional review board.

### Frailty assessment

We assessed frailty using a Frailty Index (FI), which was constructed following a standardized procedure [30]. The FI is based on 40 age-related multidimensional deficits (Supplementary Table 1). Any symptom, disease, functional impairment, or laboratory abnormality can be classified as a deficit and included in the FI if it meets the following criteria: (i) it is negatively associated with the individual's health status, representing a health deficit; (ii) it is age-related, with prevalence generally increasing with age; (iii) it is present in at least 1% but no more than 80% of the population sample; and (iv) it has less than 5% missing data. The selected variables were also required to fulfill the following general requirements: (i) they covered multiple organs and systems, ensuring a multidimensional approach; (ii) they included measures of functional status (e.g., disabilities) in addition to diseases and comorbidities; and (iii) they comprised a sufficient number of deficits (at least 30) to ensure the robustness of the FI estimates [17]. Each deficit within the FI was scored as follows: 0 indicated the absence of the deficit, while 1 indicated its presence. The FI was calculated as the ratio between the number of deficits experienced by the individual and the total number of considered deficits (i.e., 40). Thus, the FI ranged from 0 (indicating no deficits) to 1 (indicating all deficits). For example, if a participant exhibited 8 out of 40 possible deficits, their FI score would be 8/40 = 0.2. Validated FI cutoff points from the general population were employed to classify participants into three categories: relatively fit (FI ≤ 0.10), less fit (0.10 < FI ≤ 0.21), and frail (FI > 0.21) [31–33]. The clinical assessment was performed by a clinician blinded to the experimental procedures.

### Kinematic analysis of finger-tapping

The motor task was adopted from previous studies [9, 11, 12, 23–26, 34–38]. Participants were comfortably seated in a chair and were instructed to tap their index finger repetitively on their thumb for 15 s (finger-tapping). Three 15-s trials were recorded for each side in a randomized order. Participants were allowed to rest for 45–60 s between acquisition trials to avoid fatigue. Before starting the motor task, one practice trial was permitted for the participants to become familiar with the experimental setting. Finger-tapping movements were recorded using a 3D-optoelectronic system (SMART motion system, BTS Engineering, Milan, Italy). This system comprises three infrared cameras (sampling rate, 120 Hz) that follow the 3D displacement of reflective markers taped to the participant’s hand. We used five reflective markers (5 mm in diameter) of negligible weight. One marker was placed on the tip of the index finger, and another was put on the tip of the thumb. Three additional reflective markers were placed on the hand (one on the head of the 2nd metacarpal bone, one on the base of the 2nd metacarpal bone and one on the base of the 5th metacarpal bone) to define a reference plane that was used to mathematically exclude possible contamination due to hand tremor or other unwanted movements from the index finger recordings [21, 39]. Kinematic data were analyzed using a dedicated software that reconstructs the 3D space displacements of the reflective markers off-line (SMART Analyzer, BTS, Milan, Italy) and determines the kinematic variables of interest [9, 11, 12, 23–26, 34–38]. Specifically, we measured the total number of movements, as well as movement rhythm, represented by the coefficient of variation (CV) computed by the standard deviation/mean value of the inter-tap intervals (with higher values representing lower regularity of repetitive movements). Linear regression analysis was employed to estimate movement amplitude (degrees), movement velocity (degrees per second), as well as the progressive reduction in amplitude and velocity (i.e., sequence effect) observed across the 15-s trials [9, 11, 12, 23–26, 34–38]. The kinematic recording and analysis were performed by researchers blinded to the clinical assessment of participants.

### Statistical analysis

We divided the entire sample of participants into two subgroups (younger adults and older adults) using a median split procedure based on age [40–42]. Sex differences between younger adults and older adults were assessed using Fisher's exact test. Differences in clinical data between subgroups were evaluated with the Mann–Whitney U test. Unpaired t-tests were used to assess potential differences between subgroups for each kinematic parameter. We compared the overall FI scores between the two subgroups using the Mann–Whitney U test. We calculated the effect size using Cohen’s d for parametric tests and the rank-biserial correlation (r_rb_) for non-parametric tests. We used validated FI cutoff values to compare the percentage of frail subjects in the two subgroups using Fisher's exact test. We conducted Spearman’s correlation analysis to explore possible relationships between clinical variables (e.g., age, FAB, MMSE) and kinematic parameters in the older adults subgroup. The confidence interval (CI) for the Spearman correlation was calculated using Fisher's z-transformation, followed by the CI computation, and then reconverted to the Spearman scale. We assessed the normality of continuous variables using the Kolmogorov–Smirnov and Shapiro–Wilk tests. Unless otherwise specified, we report results as mean ± SD. We applied a significant level of P ≤ 0.05 for all statistical tests. We performed all analyses using the Statistical Package for Social Sciences (SPSS, version 25.0, IBM, New York, USA).

## Results

The subgroup of younger adults included 26 subjects (18 females, mean age ± 1 SD = 56.77 ± 4.76 years), while the older adults subgroup included 39 subjects (22 females, mean age ± 1 SD = 71.15 ± 5.54 years)**.** No significant differences in terms of sex distribution were found between subgroups (*P* = 0.22). Clinical assessment revealed slightly lower MMSE scores in the older adults compared to the younger adults subgroup (28.72 ± 1.39 vs 29.69 ± 0.74, r_rb_ = 0.442 *P* = 0.001). Similarly, FAB scores were slightly lower in older adults (17.13 ± 1.24 vs 17.58 ± 0.76, r_rb_ = 0.291, *P* = 0.037) (Table 1).

**Table 1:** Demographic and clinical data. We divided the entire sample into two subgroups (younger adults and older adults) using an age-based median split procedure**.** Age is expressed in years. F: females; MMSE: Mini-Mental State Examination; FAB: Frontal Assessment Battery. FI: frailty index. Relatively fit refers to individuals with FI ≤ 0.10, less fit to individuals with a FI > 0.1 and ≤ 0.21), while frail refers to subjects with a FI > 0.21. Results are shown as mean values ± 1 standard deviation (SD). The percentage is shown in round brackets. P values from Mann–Whitney U test. Significant values are shown in bold

	Whole sample	Younger adults	Older adults	P-value
N°	65	26	39	
Age	65.40 ± 8.80	56.77 ± 4.76	71.15 ± 5.54	** < 0.001**
Sex	40 F (61.5%)	18 F (69.2%)	22 F (56.4)	0.22
MMSE	29.11 ± 1.27	29.69 ± 0.74	28.72 ± 1.39	**0.001**
FAB	17.31 ± 1.09	17.58 ± 0.76	17.13 ± 1.24	**0.037**
FI	0.10 ± 0.07	0.10 ± 0.07	0.11 ± 0.07	0.341
*Relatively Fit*	42 (64.6%)	19 (73.1%)	23 (59%)	0.18
*Less fit*	18 (27.7%)	5 (19.2%)	13 (33.3%)	0.17
*Frail*	5 (7.7%)	2 (7.7%)	3 (7.7%)	0.67

### Frailty assessment

When considering the whole sample, we found that FI scores ranged between 0.00 and 0.325 and had a characteristic right-skewed distribution. Median and mean FI values were 0.1 (interquartile range 0.1) and 0.1 (SD 0.07), respectively. Female participants had higher FI scores compared to males (0.12 ± 0.07 vs 0.08 ± 0.06; r_rb_ = 0.334, *P* = 0.02). Overall, 5 subjects (7.7% of the total sample) were classified as frail (FI > 0.21), 18 individuals (27.7%) as less fit (0.10 < FI ≤ 0.21), and 42 (64.6%) as relatively fit (FI ≤ 0.10). When analyzing the two subgroups, we observed comparable FI scores between younger and older adults (0.10 ± 0.07 vs 0.11 ± 0.07, *P* = 0.34). The percentage of frail subjects was comparable between subgroups [2 frail subjects (7.7%) in the younger adults subgroup, 3 (7.8%) in the older adults subgroup, *P* = 0.67; 5 (19.2%) less fit individuals in the younger adults subgroup, 13 (33.3%) in the older adults subgroup, *P* = 0.17; 19 (73.1%) relatively fit individuals in the younger adults subgroup, 23 (59%) in the older adults subgroup, *P* = 0.18].

### Kinematic analysis of finger-tapping

**Fig. 1:**
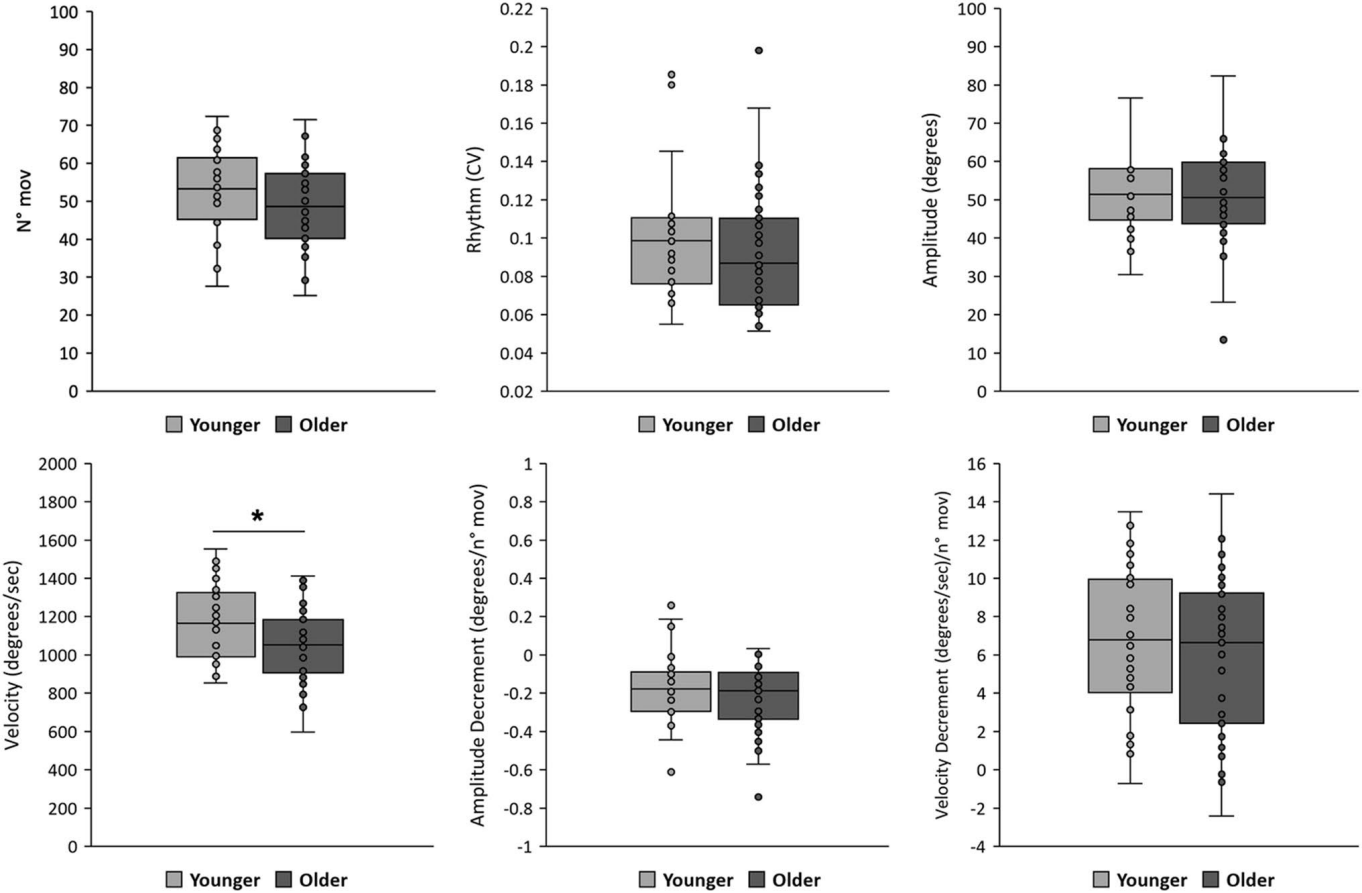
Kinematic variables of repetitive finger-tapping. N° mov.: number of movements; CV: coefficient of variation. Light grey: younger adults subgroup. Dark grey: older adults subgroup. Horizontal lines indicate the median values, boxes indicate ± 1 standard error (SE) of the mean, and whiskers indicate ± 1 standard deviation (SD) of the mean. Circles indicate each individual value in the two subgroups. Asterisk indicates *P* < 0.05 from unpaired t-test

A preliminary analysis showed no differences in terms of kinematic parameters between the dominant and non-dominant sides (all *P* > 0.05 by paired t-test, Supplementary Table 2). The average values from the two sides were used for further analysis. Kinematic results are presented in Fig. 1. We found significant differences in terms of movement velocity during finger-tapping between subgroups, with lower values in older adults (younger adults: 1170.54 ± 203.19 degrees vs older adults: 1046.33 ± 187.84; Cohen’s d = 0.640, *P* = 0.014). Conversely, other kinematic parameters were comparable between subgroups (all *P* values > 0.05) (Fig. 1).

### Correlation analysis

**Fig. 2:**
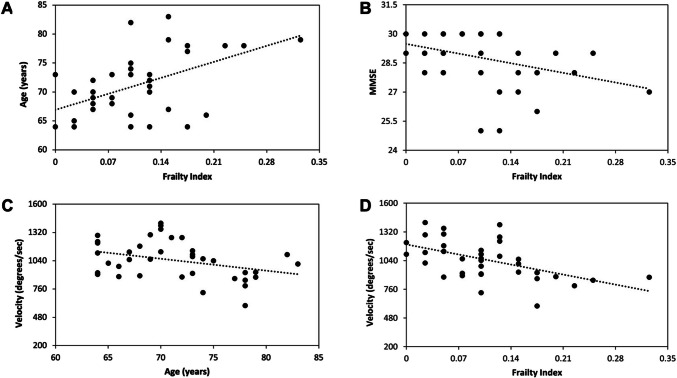
Correlations between clinical and kinematic data in older adults subgroup. **A** Frailty Index (x-axis) and age (y-axis) (*ρ* = 0.466, *P* = 0.003). **B** Frailty index (x-axis) and Mini-Mental State Examination (MMSE) (y-axis) (*ρ* = −0.388, *P* = 0.015). **C** Age (x-axis) and movement velocity (y-axis) (*ρ* = −0.335, *P* = 0.037). Age was also significantly correlated with amplitude decrement (*ρ* = −0.378, *P* = 0.018, not shown in the figure). **D** Frailty Index (x-axis) and movement velocity (y-axis) (*ρ* = −0.536, *P* < 0.001)

We found a positive correlation between age and FI (*ρ* = 0.466 [95% CI 0.18 – 0.68]; *P* = 0.003) (Fig. 2). Also, we found a negative correlation between FI and MMSE scores (*ρ* = −0.388 [95% CI −0.63—−0.08]; *P* = 0.015). Age significantly correlated with movement velocity (*ρ* = −0.335 [95% CI −0.59—−0.02]; *P* = 0.037) and amplitude decrement (*ρ* = −0.378 [95% CI - 0.62—−0.07]; P = 0.018). Again, FI showed a negative correlation with finger-tapping velocity (*ρ* = −0.536 [95% CI −0.73—−0.265]; *P* < 0.001) (Fig. 2), indicating that greater frailty is associated with more impaired motor performance. We did not find any other significant correlations between clinical and kinematic data. The correlation analysis for the younger adults subgroup is available in the supplementary materials.

## Discussion

In this study, we investigated the effects of aging on motor functions as assessed by kinematic techniques. Furthermore, we assessed the relationship between motor performance and frailty, as measured by the validated FI. Notably, we found that older adults exhibited reduced velocity in voluntary repetitive movements compared to healthy younger adult controls. Furthermore, movement velocity was negatively correlated with FI. These findings offer novel insights into the complex interplay of aging, highlighting movement velocity as a potential marker of vulnerability. This marker is closely related to frailty, a measure of deficit accumulation that reflects functional status and biological age. By combining kinematic parameters with the FI, this study sheds light on the connections between physical frailty, cognitive performance, and motor function in aging.

To exclude possible confounding factors, all study participants underwent a thorough clinical evaluation and a comprehensive neurological examination to rule out any neurological disorders, including clinically evident parkinsonism [21, 22]. Additionally, individuals with severe psychiatric conditions or those undergoing treatments that could affect the central nervous system were excluded to minimize potential biases. Clinical assessments were conducted by operators blinded to the experimental procedures, while kinematic evaluations were performed by operators unaware of the participants' clinical status, ensuring greater objectivity. To further reduce variability, all assessments were carried out under identical experimental conditions and at the same time of day. Finally, for the evaluation of motor function, we employed the finger-tapping task, a well-established tool in clinical practice [8, 11, 13, 22, 43], using standardized kinematic methods validated in numerous previous studies [9, 11, 12, 23–26, 34–38].

The first notable result from the study emerged from the kinematic analysis of finger-tapping. The age-based split analysis revealed significant differences in finger-tapping speed between younger adults and older adults subjects, with older individuals showing reduced movement velocity. We also found that age was correlated with movement velocity, as well as with a progressive reduction in amplitude during movement, meaning older individuals had a higher sequence effect during finger tapping. The human brain undergoes various changes due to physiological aging, including brain atrophy, small vessel disease, and the buildup of misfolded proteins like α-synuclein, tau, and amyloid-β peptide [2]. These changes have been observed in the brains of older individuals who are cognitively normal and relatively healthy and they manifest through modifications at the cellular and molecular levels. Hence, as aging is associated with a gradual decline in the cerebral dopaminergic system in mice [44] and humans [45, 46], the decline in motor performance in older adults may be linked to age-related reductions in dopamine levels. Dopaminergic tone is crucial for motor control, and even subtle reductions can impair voluntary movement execution. Evidence from studies on essential tremor (ET) further supports this hypothesis [35]. ET patients, often exhibiting slowed finger-tapping, may show subtle dopaminergic changes in the striatum, as indicated by reduced dopamine transporter availability, supporting a potential link between motor slowness and dopaminergic deficits. All these findings support the hypothesis that slowed finger-tapping speed in older adults may reflect similar age-related declines in dopaminergic function. Accordingly, recent neuroimaging studies, including longitudinal reports, showed structural and functional changes in the brains of individuals exhibiting subtle parkinsonian signs such as motor slowness, particularly within motor-related regions and dopaminergic pathways.^46–49^ Subtle changes in dopamine tone with aging may, therefore, disrupt motor pathways, underscoring the importance of dopaminergic modulation in maintaining fine motor control.

A second key finding from the study is that in older adults, there was a correlation between movement velocity and the FI, suggesting that frailty may be another factor, alongside aging, that contributes to the deterioration of motor performance. This finding aligns with numerous studies documenting the relationship between aging and frailty, emphasizing the progressive nature of frailty in older adults [47]. Frailty is a multidimensional syndrome and recent research emphasizes the pivotal roles of chronic inflammation [48–50] and oxidative stress [51, 52]. These processes are not only central to the aging process but also exacerbate frailty by disrupting cellular and tissue homeostasis. Chronic inflammation, often referred to as "inflammaging," is characterized by a persistent low-grade inflammatory state, marked by elevated levels of pro-inflammatory cytokines such as TNF-α and IL-6. This inflammatory environment accelerates tissue damage, impairs immune function, and contributes to muscle wasting, further diminishing resilience and increasing frailty [53]. Similarly, oxidative stress, which results from an imbalance between reactive oxygen species (ROS) and the body’s antioxidant defenses, accelerates cellular damage and dysfunction, affecting multiple organ systems, including skeletal muscle and the central nervous system. Notably, the presence of multiple comorbidities has been correlated with changes in CSF biomarkers, including elevated lactate levels [54]. Increased CSF lactate has been associated with mitochondrial dysfunction and oxidative stress, further reinforcing the connection between frailty and neurodegeneration [54]. Crucially, both chronic inflammation and oxidative stress are linked to dopaminergic dysfunction, which underlies many of the physical and cognitive impairments observed in frail individuals. Dopaminergic systems, particularly those within the striatum and substantia nigra, are highly vulnerable to oxidative damage due to their high metabolic activity and iron content, which catalyzes ROS formation [51, 55, 56]. This vulnerability, combined with the chronic inflammatory state, contributes to dopaminergic neuron loss, leading to a decline in dopamine levels. As dopamine is essential for motor control, the loss of dopaminergic function is associated with a reduction in motor performance, including impaired coordination, bradykinesia, and overall motor slowness, which are common symptoms of frailty. Neuroimaging studies have shown that frail individuals exhibit reduced activation of the primary motor cortex (M1) and altered functional connectivity between M1, the premotor cortex, and the putamen [57], key structures involved in movement planning and execution [8, 13, 58, 59].The decreased activity in these regions has been associated with a reduced capacity for cortical recruitment during motor tasks, suggesting an insufficient compensatory response to neuromuscular decline associated with aging and frailty [60]. Consequently, functional connectivity alterations between the motor cortex and the basal ganglia, particularly the putamen, may contribute to motor slowing and impaired voluntary movement generation. Beyond the dopaminergic system, other neural circuits play a significant role in the motor and cognitive impairments observed in frailty. One of the critical systems involved is the cholinergic system, which is essential for motor coordination, cognitive function, and overall neural regulation. Acetylcholine modulates motor cortex and basal ganglia activity, influencing both movement execution and synaptic plasticity [61]. Neuroimaging studies have shown a reduction in cholinergic innervation, particularly in the nucleus basalis of Meynert, which correlates with motor and cognitive deficits [62]. Additionally, the cholinergic system interacts with cortico-striatal circuits to modulate dopaminergic transmission. Disruptions in this balance may contribute to motor slowness and reduced motor initiative observed in frailty subjects [63]. This aligns with previous neurophysiological studies demonstrating a link between motor slowing and cholinergic dysfunction in M1 in dementia [64]. Structural brain alterations have been implicated in the pathophysiology of frailty, with evidence suggesting that cerebrovascular damage [65], gray matter atrophy [66, 67], white matter abnormalities [68, 69], and altered connectivity of cerebral areas, such supplementary motor area [70] contribute to this condition. Additionally, volumetric losses in the cerebellum, hippocampus, and middle frontal medial cortex have also been observed [66]. However, cerebellar volumetric loss appears to be particularly critical. Strong connections between the cerebellum and basal ganglia, such as the cerebello-nigrostriatal pathway, are well-known. Cerebellar projections to the substantia nigra pars compacta play a prominent role in the dopaminergic modulation of the basal ganglia and may constitute an additional pathway through which the cerebellum contributes to movement execution [71–73]. This perspective aligns with our findings, which demonstrated a negative correlation between movement velocity and frailty, emphasizing that both cerebellar alterations and reduced dopaminergic tone contribute to this phenomenon. The cerebellum's critical role in modulating movement vigor, combined with the decreased dopaminergic input to the basal ganglia, creates a dual mechanism underlying the impaired motor performance observed in frail individuals.

Additionally, the negative correlation between frailty and cognitive performance, as measured by the MMSE, emphasizes the broader implications of frailty on cognitive function. This finding is consistent with previous research [14, 15] and aligns with evidence suggesting that frailty reflects underlying neurodegenerative processes that impair executive function, memory, and cognitive flexibility [45, 74]. The association between frailty and cognitive decline underscores the importance of considering frailty not only as a marker of physical health but also as a key indicator of cognitive resilience in aging populations. Given the intertwined nature of motor and cognitive functions in aging, frailty offers valuable insights into the complex interplay between these domains and may serve as a critical target for interventions aimed at mitigating age-related declines in both physical and cognitive health. Together, these findings suggest that frailty integrates cognitive and motor dimensions of aging through shared underlying mechanisms. The interplay between motor impairment, frailty, and cognitive decline possibly involves systemic inflammation, oxidative stress, and vascular changes, which disrupt both central and peripheral nervous system function. Frailty thus emerges as a critical indicator of neurophysiological aging, with implications for targeted interventions aimed at mitigating age-related decline.

The results of the present study are consistent with the observation that mild parkinsonian signs (MPS) are commonly observed in older adults. Prevalence estimates range from 15 to 95%, increasing with advancing age [3, 5, 75, 76]. MPS can be categorized into four primary domains: bradykinesia, rigidity, tremor, and postural or gait impairment [77, 78]. Although individuals with MPS do not meet the established diagnostic criteria for PD [21, 79], they may display subtle symptoms across one or more of these domains [6, 75, 80–82]. Importantly, unlike PD patients, individuals with MPS are less likely to exhibit rest tremor and typically show poor responsiveness to levodopa [6, 78, 83, 84]. MPS are progressive and linked to significant functional impairments, including a marked decline in autonomy in daily living activities [81, 85]. The presence of MPS has also been associated with an increased risk of cognitive impairment (ranging from mild cognitive impairment to dementia), PD, and mortality [86–88]. While the underlying neurophysiological mechanisms are not well understood, they are believed to involve multifactorial processes, including age-related declines in dopaminergic activity, vascular pathologies, and neurodegenerative conditions such as PD and AD [5, 6, 80].

This study has several limitations that should be acknowledged. First, the sample size was relatively small. This limited sample size may reduce the generalizability of the findings to broader populations. Second, the proportion of participants classified as frail was relatively low, potentially limiting the statistical power to detect more nuanced relationships between frailty, motor, and cognitive outcomes. Third, the study population consisted of healthy community-dwelling individuals, with an underrepresentation of more vulnerable populations, such as institutionalized individuals. Future studies with larger, more heterogeneous cohorts, including individuals with different levels of frailty, are warranted to strengthen the robustness of these findings. Finally, the cross-sectional design of the study, while useful for identifying associations, does not allow for causal inferences regarding the relationships between aging, frailty, and motor decline. Longitudinal studies are necessary to determine the directionality of these relationships and to assess the progression of motor impairment over time in relation to frailty status.

## Conclusions

This study highlights the significant associations between aging, frailty and motor function in healthy adults. Older adults exhibited reduced movement velocity compared to younger adults, with frailty emerging as a key factor linked to these declines. Specifically, frailty was negatively correlated with MMSE scores, reflecting its impact on cognitive performance and motor execution. The relationship between frailty and movement velocity represents a novel contribution, suggesting that motor performance may serve as a sensitive indicator of physical vulnerability in aging populations. Furthermore, the study highlights the importance of assessing frailty in healthy adults to identify those at risk of not only cognitive but also motor decline. Future research should focus on larger and more diverse populations to validate these findings and explore causal mechanisms underlying the interplay between frailty, cognitive, and motor domains. Longitudinal studies are needed to investigate the predictive value of frailty measures in age-related neurophysiological decline and to develop targeted interventions to mitigate these effects.

## Supplementary Information

Below is the link to the electronic supplementary material.

Supplementary file1 (DOCX 19 KB)

## Data Availability

The datasets generated during and/or analysed during the current study are available from the corresponding author on reasonable request.

## Abbreviations

AD: Alzheimer’s Disease
CV: Coefficient of Variation
CI: Confidence Interval
ET: Essential Tremor
FI: Frailty Index
FAB: Frontal Assessment Battery
MMSE: Mini-Mental State Examination
MPS: Mild Parkinsonian Signs
PD: Parkinson’s Disease
M1: Primary Motor Cortex
r_rb_: Rank-biserial correlation
ROS: Reactive Oxygen Species
SD: Standard Deviation
