# Supplementary material for: Aging, frailty, and their effects on motor performance: evidence from kinematic analysis
Source: Neurol Sci. 2025 Mar 18;46(7):3097–106. doi: 10.1007/s10072-025-08092-z (PMC12152081; doi:10.1007/s10072-025-08092-z)
Supplement: Supplementary file 1 — Supplementary file1 (DOCX 19 KB) [file 10072_2025_8092_MOESM1_ESM.docx]

**AGING, FRAILTY, AND THEIR EFFECTS ON MOTOR PERFORMANCE: EVIDENCE FROM KINEMATIC ANALYSIS**

Martina De Riggi, MD^1^, Giulia Paparella, MD, PhD^1,2^, Antonio Cannavacciuolo, MD^2^, Martina Salzillo, MD^1^, Filippo Nuti, MD^1^, Ludovica Quarta, MD^3^, Daniele Birreci, MD^1^, Davide Costa, MD^2^, Luca Angelini, MD^2^, Marco Canevelli, MD, PhD^1^, Matteo Bologna, MD, PhD*^1,2^

*^1^Department of Human Neurosciences, Sapienza University of Rome, Italy*

*^2^IRCCS Neuromed Pozzilli (IS), Italy*

*^3^Department of Clinical Internal, Anesthesiologic and Cardiovascular Sciences, Sapienza University of Rome, Rome, Italy*

**Corresponding Author:**

Prof. Matteo Bologna

Department of Human Neurosciences

Sapienza University of Rome

Viale dell’Università, 30, 00185 Rome, Italy

E-mail: [matteo.bologna@uniroma1.it](mailto:matteo.bologna@uniroma1.it)

| 1. Hypertension |
| --- |
| 1. Diabetes |
| 1. Arrhythmia |
| 1. Chronic heart failure |
| 1. Intestinal disorder |
| 1. Thyroid disease |
| 1. Genitourinary disease |
| 1. Cancer |
| 1. Osteoporosis |
| 1. COPD |
| 1. Renal failure |
| 1. Ischemic heart disease |
| 1. Liver disease |
| 1. Falls (in the last 6 months) |
| 1. Involuntary weight loss (≥ 4.5 kg in the last 6 months) |
| 1. Hearing impairment |
| 1. Declivous oedema |
| 1. SpO_2_ < 94% |
| 1. HR < 60 bpm or > 90 bpm |
| 1. Obesity (BMI ≥ 30) or Underweight (BMI < 18) |
| 1. Dizziness |
| 1. Chronic pain |
| 1. Urinary urgency |
| 1. headache |
| 1. Focal neurological signs |
| 1. Dehydration |
| 1. Irritability |
| 1. Spatiotemporal disorientation 2. Dyspnea 3. Sphincter incontinence 4. Difficulty with distances > 400 m 5. Difficulty with transportation outside the home 6. Difficulty with money/finances 7. Difficulty with medications 8. Difficulty climbing stairs 9. Hemoglobin (< 13,5 g/dL in males; <12 g/dL in females) 10. Hypoalbuminemia 11. Thrombocytopenia 12. Hypercreatininemia 13. High blood urea nitrogen levels |

**Supplementary Table. 1** **Items included in the computation of the 40-items Frailty Index (FI).** COPD: Chronic obstructive pulmonary disease; SpO_2_: peripheral oxygen saturation; HR: heart rate; BMI: Body Mass Index.

|  | | | Average | | | Dom. Side | | | Non-Dom. Side | | | P-value | | |
| --- | --- | --- | --- | --- | --- | --- | --- | --- | --- | --- | --- | --- | --- | --- |
| Number of Mov. | | | 50.30±11.63 | | | | 51.49±12.52 | | | 49.12±12.90 | | | | 0.067 |
| CV | | | 0.10±0.03 | | | | 0.09±0.04 | | | 0.10±0.04 | | | | 0.082 |
| Amplitude | | | 51.17±11.24 | | | | 52.30±13.78 | | | 50.04±14.16 | | | | 0.524 |
| Velocity | | | 1112.65±225.77 | | | | 1112.65±225.77 | | | 1079.37±267.21 | | | | 0.276 |
| Ampl. decrement | | | -0.20±0.18 | | | | -0.21±0.24 | | | -0.19±0.21 | | | | 0.099 |
| Vel. decrement | | | 5.67±5.19 | | | | 5.67±5.19 | | | 6.98±4.97 | | | | 0.351 |
|  |  | |  |  | | |  | | |  | |  |  |  |

**Supplementary Table 2. Kinematic variables of dominant and non-dominant side in the whole sample.** CV: coefficient of variation. Amplitude is expressed in degrees. Velocity is expressed in degrees per second. Amplitude decrement is expressed in degree per number of movements. Velocity decrement is expressed in (degrees per second/number of movements). Results are shown as mean values ± 1 standard deviation (SD). P values from paired t-tests. Significant values are shown in bold.

**Supplementary Data: Correlation Analysis in the younger adults subgroup**

We conducted Spearman’s correlation analysis to explore possible relationships between clinical variables (e.g., age, FAB, MMSE) and kinematic parameters in the younger adults subgroup. We found a positive correlation between age and FI (ρ = 0.499 [95% CI 0.14 - 0,74]; P = 0.009). We did not find any other significant correlations between clinical and kinematic data.
